# Supplementary figures and images for: New Nitric Oxide-Releasing Compounds as Promising Anti-Bladder Cancer Drugs
Source: Biomedicines. 2023 Jan 12;11(1):199. doi: 10.3390/biomedicines11010199 (PMC9855963; doi:10.3390/biomedicines11010199)

# Supplementary material

## $^1\text{H}$ and $^{13}\text{C}$ -NMR spectra

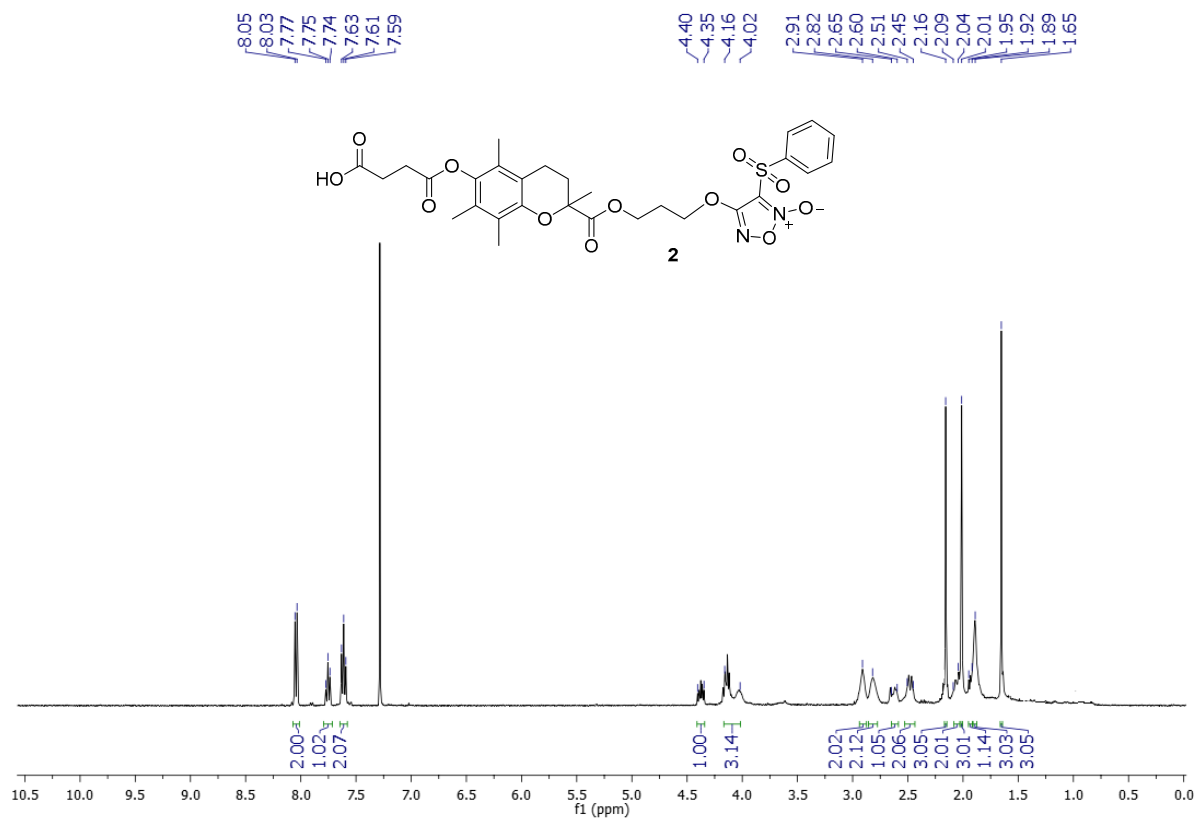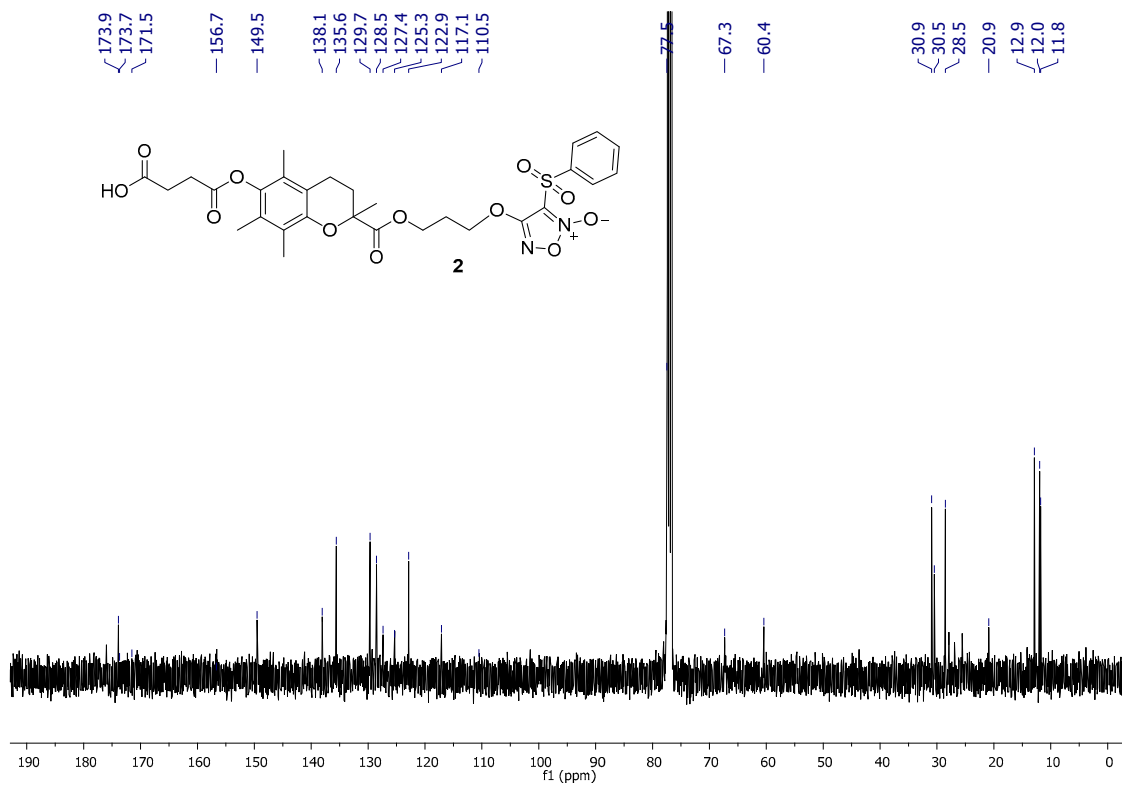

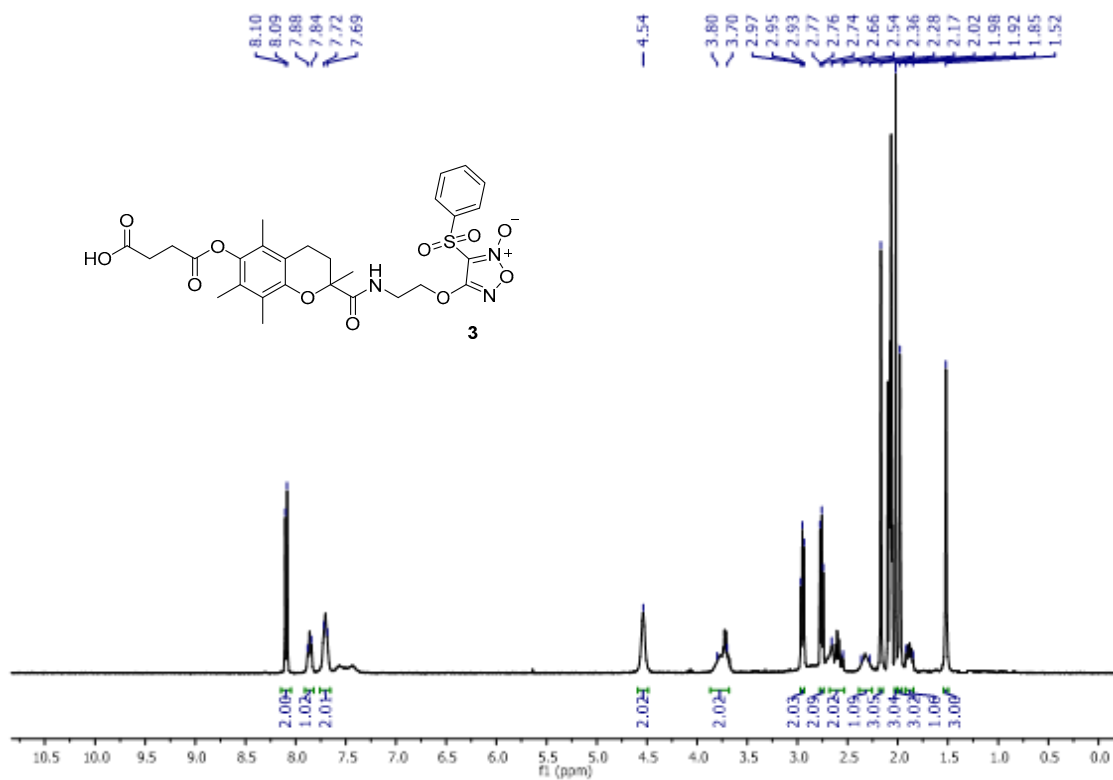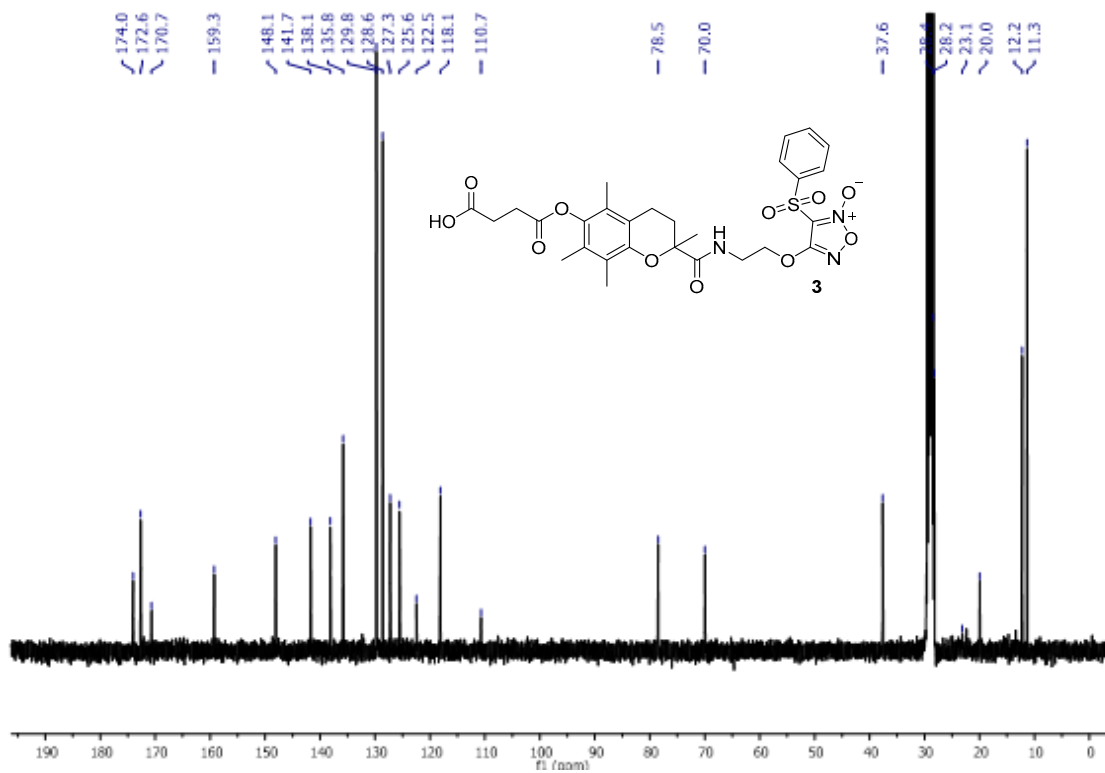

Supplement: Supplementary file 1 [file biomedicines-11-00199-s001.zip › biomedicines-2147650-supplementary.pdf]
